# Attention to text in video predicts young children's orthographic knowledge

**DOI:** 10.1111/bjep.70049

**Published:** 2025-11-26

**Authors:** Tanya Kaefer, Susan B. Neuman

**Affiliations:** ^1^ Faculty of Education Lakehead University Orillia Ontario Canada; ^2^ Steinhardt School of Culture, Education and Human Development New York University New York New York USA

**Keywords:** contexts of learning, digital technologies and learning, literacy—reading, pre‐school education, support for learning, types of learning

## Abstract

**Background and Aims:**

This study examined preschool‐aged children's attention to text in video, and whether it may be related to their developing orthographic knowledge.

**Sample 1:**

Study 1 showed 66 children videos that included text.

**Method**

Children's attention to the video was measured using eye‐tracking, and their recognition of orthographic patterns within the video was tested after viewing.

**Results**

During viewing, children attended to the text 6% of the time it was available. However, children who both had pre‐existing letter knowledge and attended to the text in the video were able to identify the written words.

**Sample 2:**

A second study extended these findings to a younger age group (*n* = 59).

**Method:**

In Study 2, we also showed children an unrelated storybook that incorporated the target words from the videos and measured attention to that storybook.

**Results:**

Results again showed little attention to text, but some recognition of written words for those who did attend. Study 2 also showed that children who recognized the written words from the video attended more to those words in a different context.

**Conclusion:**

Overall results suggest a relationship between letter knowledge, attention and developing orthographic knowledge.

## INTRODUCTION

Parents, teachers and policy makers have become increasingly aware of broad‐spectrum failures in reading instruction for children. In the United Kingdom, 26% of children at the end of year 6 are not meeting the expected standard of reading (UK Department for Education, 2024). In the United States, the numbers are worse, with 37% of children in grade 4 unable to read at a basic level (National Centre for Education Statistics, 2022), a number that has increased from the already alarming rate of 34% pre‐pandemic (National Centre for Education Statistics, 2019). These low reading rates have put a spotlight on how early reading develops, and what factors can influence children's early literacy skills.

Although science has largely converged around structured literacy—including systematic phonics instruction (e.g., Ehri, 2020) and the importance of background knowledge for comprehension (e.g., Pinkham et al., 2012)—an additional factor that has received less attention is children's orthographic knowledge. Orthographic knowledge encompasses children's understanding of print, both generalized understanding of common patterns within written language (sublexical orthographic knowledge) as well as the actual spelling of particular words (lexical orthographic knowledge) (Apel et al., 2019; Conrad & Deacon, 2023). Children's knowledge of print prior to direct instruction influences their literacy development throughout the elementary years (LaParo & Pianta, 2000; Rothe et al., 2024), thereby making early orthographic knowledge a crucial issue in education research. In the current study, we examined young children's understanding and recognition of lexical orthography and how it may be related to their exposure and attention to text.

The development of the ability to recognize specific orthographic patterns is not an all‐or‐nothing phenomenon. Rather, this is likely a skill that develops slowly. Ehri's stages of reading (1979, 2014) present lexical orthographic knowledge as something that develops from practice with the alphabetic principle—learning sublexical orthographic rules alongside letter‐sound correspondences allows children to slowly develop a recognition of ‘sight words’ that they can read and recognize based on the lexical orthographic pattern. Similarly, Treiman's Integration of Multiple Patterns theory (2017) focuses on statistical learning of common and allowable orthographic patterns in the language leading to first sublexical and later lexical orthographic knowledge. By contrast, Share's self‐teaching hypothesis doesn't require sublexical knowledge to develop first, and instead posits that the intensive attention to text required by tasks like phonemic recoding builds children's lexical orthographic knowledge, such that recognition of individual words is almost entirely dependent on this kind of activity (Share, 2004, 2025). In this theory, activities that enhance phonological recoding are more likely to improve orthographic knowledge (Li & Wang, 2022). One thing all these theories have in common, however, is that building lexical orthographic knowledge is dependent on experiences with print that would allow children to build this knowledge. This has been supported by some recent studies (e.g., Heintzman & Deacon, 2024; Shakory et al., 2021) which have shown that shared‐book reading may be one way that children develop orthographic knowledge. Theoretically, children who intensively attend to print are more likely to have the opportunity to build the kind of orthographic knowledge that can support further literacy and reading development.

Unfortunately, simple exposure to text may not provide children with the opportunity to intensively engage with print. Historically, teachers have used classroom read‐alouds and shared book reading as a primary way of exposing children to print in preschool and kindergarten (Damber, 2015). However, research examining children's eye movements has shown that preschool‐aged children attend to illustrations over print more than 90% of the time when engaged in shared book reading (e.g., Evans & Saint‐Aubin, 2005). Nonetheless, there is some evidence that even this brief exposure may help build orthographic knowledge (Apel et al., 2013; Shakory et al., 2021).

More recently, there has been a push towards incorporating multi‐media strategies as an engaging means of introducing text to children (Ohler, 2013), particularly for those from low‐income backgrounds (Neuman, 2014). There is some evidence that video has some natural advantages over print in guiding children's attention. In particular, the formal features of video—things like movement, sound effects and visual effects—can draw attention to relevant areas of the screen (e.g., Huston & Wright, 1983). These formal features have been shown to be consistently successful at drawing children's attention, even in infancy (e.g., Anderson & Burns, 2013). Additionally, some work with older children has suggested that text on‐screen while watching television can enhance vocabulary development (e.g., Linebarger, 2001; Linebarger et al., 2013), suggesting that textual representation may be supportive in a media context. Some work on orthographic learning has posited that visual exploration of the text is an essential requirement for orthographic learning (Ginestet et al., 2022) which would suggest that features which guide attention to text would be particularly supportive.

On the other hand, formal features work best when paired with the comprehensibility of the media. Studies have suggested that children prefer to attend to media that is understandable (Anderson & Pempek, 2005) and that children may use the formal features of media as markers of interesting or relevant information (Calvert et al., 1982), which could suggest that formal features that direct attention to incomprehensible stimuli may not be as effective. The benefit of formal features in video may not extend to information, like text, that may not contain meaningful information for preschoolers. This is supported by research using children's eye movements to gauge their attention. Children who can recognize more written words and letters fixate more on words and letters in storybooks (Evans et al., 2009), and children with larger overall vocabularies—often used as a proxy for overall background knowledge (e.g., Anderson & Freebody, 1981)—fixate more on parts of illustrations that represent novel information within the text when that novel information is referenced (Evans & Saint‐Aubin, 2013). This suggests that what children already know may guide their attention and their learning.

The goal of the current study is to explore the development of children's lexical orthographic knowledge through video. To that end, we conduct two studies in which we address the following research questions:

1. Do children attend to text in video format?
2. Do children recognize written words from the videos?
3. Is children's recognition of written words related to their pre‐existing letter knowledge?
4. Is children's recognition of written words related to their attention to those words in the videos? and
5. Do children attend more to the words introduced in the video when they later see those words in a book?

## STUDY 1

In Study 1, we address our first four research questions by showing children a commercially available video that aims to teach vocabulary by highlighting both the written forms and concepts associated with challenging words. This research was cleared for ethics by the IRB at New York University.

## METHOD

### Participants

The sample included 66 children (31 girls) recruited from 8 pre‐k (a district‐wide early childhood program for 4‐year olds) classrooms with an average age of 4 years 4 months, ranging from 3.8 to 4.6 months; 61% were African‐American and 39% Hispanic. Local schools volunteered to be part of the study through the public‐school board, and then individual children in each class provided parental consent and child assent prior to participation. Using G*power (Faul et al., 2009), we determined that our analyses, with a medium effect size and power set at .8, required 55 participants. Average income among families in this catchment area was $19,000 USD per year. All participants qualified for free and reduced lunch.

### Materials

#### Letter recognition

Children's letter knowledge was assessed using the Upper‐case Letter Recognition subtest on the Phonological Awareness Literacy Screening (PALS‐PreK) (Invernizzi et al., 2004). Children were asked to point to and identify the upper‐case letters that were listed out of order. Cronbach's alpha was .84. Children scored an average of 15.24 (*SD* 11.43) for upper‐case letters. Children were also tested on lower‐case letters, but a floor effect (50% of students scoring 0) prevented this data from having enough variability to be used as a predictor.

#### Video

Children viewed three 90‐s segments (counter‐balanced) of ‘What's the Word’, a commercially available video series produced by *Noggin*, and available through their app. These videos aimed at introducing sophisticated words both in written and oral format through rap. Each video focused on a single highly sophisticated word, which was repeated multiple times both written and orally. The combination of sophistication of the words as well as the number of repetitions in different formats made this video series an ideal material set to examine written and oral language development in a format that preschool‐aged children encounter naturally. Researchers selected individual videos based on the words presented, ensuring that the words varied in their imageability (e.g., muscle, hypothesis) and word class (e.g., cooperate). Each word was repeated 8–13 times per video, and the text was displayed during 6–8 of these repetitions. Words were shown at the top or centre of the screen, in lower‐case letters, in a child‐friendly font. Different formal features were used to highlight written words, including sound effects, movement and visual effects.

#### Text recognition

Children were given a 9‐item assessment of written words, similar in format to the PPVT. Children viewed a grid of four words on a computer screen and were asked to point to the target. Of the four words, one depicted the target and three were foils (i.e., two words from the other viewed videos, one word from a previously unseen episode). The first three items included the lower‐case word as depicted in the videos (screenshots of the text with no images or other context cues). The second three items included the word using the same font as displayed on the screen, but without the background and colours from the video, and the final three items included the word in a completely different font, with no contextual support. Participants' pointing behaviour was scored dichotomously and summed to yield an overall target word learning score, which was then converted into a proportion score. Cronbach's alpha for this measure was .79.

### Apparatus

Eye movements were measured with a Tobii Technology T120 eye‐tracker integrated into a 17 in. thin‐film transistor monitor (Psychology Software Tools, Pittsburgh, PA). This is a remote eye‐tracking system that had no contact with the child. The typical spatial accuracy of this system is approximately .5 visual degrees, and the sampling rate is 120 Hz. Head movements typically result in a temporary accuracy error of approximately .2 visual degrees. In the case of particularly fast head movements (i.e., over 25 cm/s), there is a 300 ms recovery period to full tracking ability.

### Procedure

Each child was invited by a research assistant to visit a quiet space in the school. First they completed the letter recognition task, and then they were seated approximately 60 cm from a computer monitor. All three episodes were randomized for each viewer. Video scenes were displayed on the Tobii monitor with a second monitor facing the second research assistant. Tobii Studio Professional 3.0 software was used for stimuli presentation and data processing.

To calibrate gaze, an attention grabber was shown at five points on the screen. A manual calibration procedure was used; accuracy was checked by Tobii Studio software and repeated as necessary. All children were required to pass the calibration before participating in the rest of the procedure; there was no missing data. After calibration, the child would then view three episodes in counterbalanced order. During each episode, the research assistant was able to follow the child's eye movements and behaviours using the live viewing on the second monitor. Total duration of the viewing was approximately 4.5 min. Following the viewing, the child was administered assessments and, after approximately 15 min, returned to the classroom.

### Data extraction

Areas of interest (AOIs) were drawn around the text for each scene in the video where text was available, as well as around the images associated with the vocabulary word in each scene (e.g., for cooperate, images of children working together were identified). We then extracted the total amount of visit time (a measure that includes both fixations and saccades within the AOI) to these scenes. Fixations were defined as any gaze coordinates lasting at least 60 ms and were identified using the Tobii Studio fixation filter. Adjacent gazes (i.e., gazes within a .5° radius, lasting <75 ms) were merged into a single fixation. Although visit duration can be a crude measure of engagement, including both fixations and saccades has been demonstrated as a strong measure of visual attention (Mahanama et al., 2022) and has been used in studies of children's visual attention (e.g., Donmez, 2023; Lu et al., 2021). Children's visit duration was divided by the amount of time each target was available (e.g., the amount of time spent attending to the text divided by the total amount of time the text was available on screen) to create a proportion score.

## RESULTS AND DISCUSSION

All statistical analyses were conducted using SPSS v.29. Descriptive statistics and normality tests were completed to examine children's text recognition. One‐sample *t*‐tests were used to examine whether children's performance differed from chance, and the relationship between children's attention and performance was examined using hierarchical regression (see tables for full models). Children's age‐in‐months was included as a covariate in all regression analyses. The distribution for text recognition scores showed a moderate level of skew (skew = .92) and approximately normal kurtosis (kurtosis = −.05). All analyses used were robust to this level of skew (Knief & Forstmeier, 2021); therefore no further transformations were conducted. Descriptive statistics for both studies can be found in Table 1.

**TABLE 1:** Means (and standard deviations) for Studies 1 and 2.

	Study 1	Study 2
Proportion of time spent attending to text	7% (8.5)	3% (4%)
Upper‐case letter knowledge	15.24 (11.43)	12.16 (11.54)
Text recognition (proportion correct)	.33 (.28)	.27 (.26)
Proportion of time spent attending to text in new context	N/A	3% (9%)

### Attention

Preschoolers attended to the print about 7% of the time that print was available. By contrast, portions of the screen dedicated to demonstrating the meaning of the vocabulary words were attended to much more often, about 56% of the time when presented alone, and about 66% of the time when a character demonstrated the word.

### Text recognition

When identifying the written word from the video, children were correct about 33% of the time, which was above chance, (25%), *t*(65) = 2.30, *p* = .025, *d* = 1.57. Both letter knowledge and attention to text during the video (marginally) predicted the proportion of words recognized (Table 2). There was also a significant interaction between letter knowledge and attention (Table 2). To explore the significant interaction, we conducted two additional hierarchical regressions, examining children with high and low letter knowledge separately. These analyses showed that for children with high letter knowledge, there was a significant relationship between attention to the text and word recognition, whereas for children with low letter knowledge, there was no significant relationship (see Table 3). This suggests that children who both knew more letters and attended more to the text on‐screen were more likely to recognize the written words.

**TABLE 2:** Hierarchical regression analysis of attention and letter knowledge predicting children's written word recognition in Study 1.

Step	Δ*R* ^2^	*B* ^a^	*t*‐value	*p*‐value	*sr* ^2^ ^b^
Step 1	.30			<.001	
Age in months		.26	2.32	.024	.06
Proportion of time on text		.22	1.99	.052	.04
Letter knowledge		.39	3.48	<.001	.15
Step 2	.14			<.001	
Time on text X letter knowledge		.57	3.71	<.001	.14

^a^ Standardized regression coefficient.

^b^ Squared semi‐partial correlation.

**TABLE 3:** Regression analysis of attention predicting children's written word recognition, by letter knowledge.

	*B* ^a^	*t*‐value	*p*‐value	*sr* ^2^ ^b^
Low letter knowledge				
Age in months	.49	3.00	.006	.23
Proportion of time on text	−.11	.64	.526	.01
High letter knowledge				
Age in months	.16	1.15	.260	.03
Proportion of time on text	.64	4.49	<.001	.41

^a^ Standardized regression coefficient.

^b^ Squared semi‐partial correlation.

## STUDY 2

Study 1 showed some evidence that, although children did not attend much to the text, those who both had prior letter knowledge and attended to the text may have recognized the orthographic patterns of the words. In the second study, we aimed to extend these findings by testing a new group of words, and a slightly younger group of students who had spent less time in a formal education setting. Additionally, to address the fifth research question, we added a storybook assessment that examined whether experience with the video may relate to children's attention to print in a new context.

## METHOD

### Participants

Fifty‐nine children (27 girls) with an average age of 4 years 0 months (range 2.9–4.75) were recruited from 3K (a district‐wide early childhood program for 3‐year olds) and PreK classrooms in two early learning public schools located in a high‐poverty district within a large urban city. Although this sample size is lower than Study 1, it still exceeds the minimum requirements for power as outlined in Study 1. As in Study 1, schools volunteered to participate through the public‐school board, with individual participants providing parental consent and student assent. Average income among families in this catchment area was $19,000 per year. 53% of participants were African‐American, 33% Hispanic, 8% Middle Eastern and 6% other. All children qualified for free and reduced lunch.

### Materials

The videos, letter knowledge and text recognition tests were identical to Study 1 but included different words. As in Study 1, the words varied in imageability (e.g., considerate, dilemma) and word class (e.g., persevere). An additional assessment was added to measure children's attention to text after exposure to the videos. In this assessment, children were read a brief story, containing entirely new characters and plotlines that incorporated the novel words. A Research Assistant read this story out loud while children viewed the storybook pages on a screen, to allow for measurement of eye movements. The storybook included no overlap of characters or story with the videos. Children's attention to the text and illustrations was measured.

### Apparatus

Between studies 1 and 2, we upgraded the eye‐tracking apparatus for improved accuracy and transportability. Eye movements were measured with a Tobii Pro Fusion eye‐tracker (Psychology Software Tools, Pittsburgh, PA). This is a remote eye‐tracking system that had no contact with the child. The typical spatial accuracy of this system is approximately .3 visual degrees, and the sampling rate is 250 Hz. Head movements typically result in a temporary accuracy error of approximately .2 visual degrees. In the case of particularly fast head movements (i.e., over 25 cm/s), there is a 250 ms recovery period to full tracking ability. An embedded camera is also used to record the child's reactions.

### Procedure and data analysis

The procedure was identical to the first study, with the additional assessment at the end. AOIs and fixations were created using the same definitions and format as Study 1.

## RESULTS AND DISCUSSION

The same analytical approach was taken as with Study 1. In this study, the distribution for text recognition scores was normal (skew = .16) as was kurtosis (kurtosis = −.08); no further transformations were conducted.

### Attention to text in video

As in Study 1, children attended primarily to the images. Children attended to the portions of the screen showing images of the meaning of words 66% of the time that they were available, and if a character was demonstrating the meaning of the word, they attended about 76% of the time. Children rarely attended to text, looking at those sections only about 3% of the time.

### Written word recognition

When identifying the written word from the video, children were correct about 27% of the time, which was not significantly above chance (25%), *t*(58) = .73, *p* = .479, *d* = .095. Although letter knowledge and attention to text were significantly correlated (*r* = .34, *p* = .011), and letter knowledge was similarly marginally significantly correlated with written word recognition (*r* = .25, *p* = .054), when entered into a regression together, only attention to text predicted children's recognition of the orthographic patterns (see Table 4). These results suggest that even though letter knowledge may be less of a factor for these younger children, attention to text in the videos is still related to written word recognition.

**TABLE 4:** Hierarchical regression analysis of attention and letter knowledge predicting children's written word recognition in Study 2.

Step	Δ*R* ^2^	*B* ^a^	*t*‐value	*p*‐value	*sr* ^2^ ^b^
Step 1	.17			.027	
Age in months		.07	.463	.645	.003
Proportion of time on text		.32	2.25	.029	.04
Letter knowledge		.12	.77	.443	.11
Step 2	.05			.080	
Time on text X letter knowledge		.701	1.79	.080	.18

^a^ Standardized regression coefficient.

^b^ Squared semi‐partial correlation.

### Attention to text in new context

**FIGURE 1:**
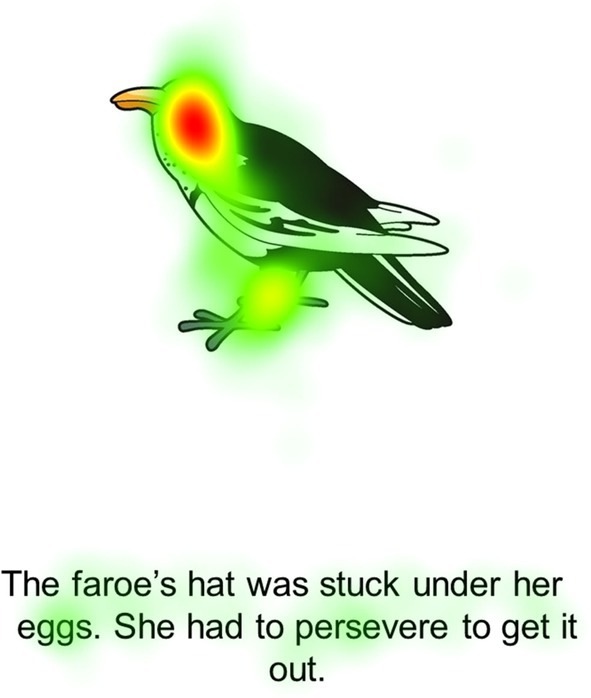
Heat map of storybook assessment page.

Children's division of attention within the new storybook was similar to that of the video, with children spending more time attending to the image (33%) than the text (2.8%). Although children spent <3% of their time looking at the text, within that, 32% of that time was spent looking specifically at the word introduced in the video (see Figure 1). This may suggest that children recognize the orthographic pattern, even in a new context.

To explore that finding further, we used hierarchical regression to examine whether children's attention to the text in the original videos, and recognition of the text in the original video, predicted their attention to text during the storybook assessment. We found that children's attention to text in the video significantly predicted their attention to text in the storybook, but this effect was entirely mediated by their recognition of the text pattern (see Table 5). This may suggest a pattern in which children's previous letter knowledge predicted their attention to text in videos, which predicted their recognition of a particular orthographic pattern, which predicted their attention to text in an unrelated storybook.

**TABLE 5:** Hierarchical regression analysis of letter knowledge, attention to text in video and recognition of text predicting children's attention to text when reading a storybook.

Step	Δ*R* ^2^	*B* ^a^	*t*‐value	*p*‐value	*sr* ^2^ ^b^
Step 1	.07			.055	
Letter knowledge		.265	1.96	.055	.07
Step 2	.08			.039	
Letter knowledge		.159	1.07	.684	.02
Attention to text video		.297	2.13	.039	.08
Step 3	.33			<.001	
Letter knowledge		.076	.684	.497	.01
Attention to text video		.092	.790	.433	.01
Word recognition		.625	5.56	<.001	.33

^a^ Standardized regression coefficient.

^b^ Squared semi‐partial correlation.

## OVERALL DISCUSSION

Taken together, these two studies show that despite the attention directing properties of the formal features, children rarely attend to text in videos—but this attention may still be related to their developing orthographic knowledge. In Study 1, children attended to the text about 6% of the time and in Study 2 even less often—about 3% of the time (although it should be noted these children were younger). These numbers are very similar to previous research with storybooks, which have shown children attending to text <10% of the time (e.g., Evans & Saint‐Aubin, 2005), suggesting that text in video operates similar to text in storybooks. In this case, the formal features of videos may not provide additional support for attention to text.

However, we also found that some children did recognize the written words after viewing the videos. This suggests that even minimal attention may be related to orthographic knowledge development. Specifically, in both studies, children who had more pre‐existing letter knowledge were more attentive to the videos, and that attention to the videos was significantly connected to their recognition of the written words. These results support the view that formal features within videos work with comprehensibility, rather than independently. When written language has some comprehensibility (i.e., when children have some knowledge of letters), then it draws more attention and, most importantly, leads to text recognition.

In Study 2, we also found that children who attended to the words in the video and recognized them in the assessments were more attentive to those words in a novel storybook context. This may begin to suggest a mechanism by which videos like these may facilitate orthographic knowledge building in the preschool years. Children who know more about letters attend more to the words on screen, which makes them more likely to recognize those words out‐of‐context, and then attend more to those words in a new context. This could indicate that attention to text builds orthographic knowledge cumulatively. Each exposure to the text, while minimal, adds a little more understanding, which, in turn, guides more attention and builds even more understanding. This is consistent with theories of orthographic development (e.g., Ehri, 1979; Li & Wang, 2022; Share, 1995) which emphasize the importance of exposure for developing orthographic knowledge. Future research is necessary to fully elucidate this developmental process.

These results suggest that despite the minimal attention paid to text that appears in videos, that text does facilitate recognition for children who already know some letters. In this way, attention to text in video offers considerable ‘bang for the buck’—even minimal attention seems to be related to orthographic knowledge. This finding is consistent with some research in children's attention to storybooks (Apel et al., 2013; Shakory et al., 2021) and would suggest that guiding children's attention to the text may be an important strategy. If that cannot be accomplished through exposure to videos with formal features that guide attention, then other attention‐directing strategies should be explored.

There are also some important limitations to the current study. First, the videos used were commercially available products; they were not designed for research. This means that the researchers had no control over word selection, the number of words presented in each video, or how the words were represented in each video. It is, however, reflective of real‐world viewing opportunities and the types of videos available to children. Further research is needed to explore whether the findings of this study can be replicated with other types of videos. Similarly, the nature of these videos only allowed for children to view a few videos at a time, limiting the words they were exposed to during the session. The similarity of findings between the two studies (and two sets of videos) suggests that these findings may replicate across multiple words, but additional research would confirm that. Additionally, these data are largely correlational in nature, and any interpretation should be cautious about drawing causal conclusions. It is possible that children with greater letter knowledge were simply able to make better guesses as to which word was which. The results from Study 2 in which students disproportionately attended to the target words in a new story would argue against this interpretation—if children were just guessing based on their overall letter knowledge, we would expect them to attend to each of the words in the new book, rather than focusing on the target word—but there is not enough evidence for a causal argument.

Children's orthographic knowledge is essential for literacy development. The findings from the current study are an important first step in understanding how text in video may be one factor that helps develop orthographic knowledge for young children. While this study offers additional support to research that suggests children spend most of their attention on pictures, the little that they spend on the printed word is important and predictive of their recognition of orthographic patterns. As many children struggle with literacy and early reading skills, understanding how orthographic knowledge develops in preschoolers is essential to understanding how to improve literacy and teaching for all children.

## AUTHOR CONTRIBUTIONS

**Tanya Kaefer:** Conceptualization; investigation; writing – original draft; visualization; writing – review and editing; methodology; data curation; formal analysis; validation. **Susan B. Neuman:** Conceptualization; investigation; funding acquisition; methodology; writing – review and editing; data curation; resources; project administration.

## CONFLICT OF INTEREST STATEMENT

The authors declare no conflicts of interest.

## Data Availability

The data that support the findings of this study are available from the corresponding author upon reasonable request.
